# Development and Feasibility of a Mixed Virtual and in‐Presence Therapeutic Education Program for Early Multiple System Atrophy Patients

**DOI:** 10.1002/mdc3.70283

**Published:** 2025-08-06

**Authors:** Margherita Fabbri, Estelle Harroch, Eva Camgrand, Paulo André Dias Bastos, Wassilios G. Meissner, Marie Christine Llorca, Cecile Travere, Marc Kermorgant, Olivier Rascol, Clémence Leung, Aude Roy, Anne Pavy‐Le‐Traon, Christine Mohara, Charlotte Scotto D'Apollonia, David Bendetowicz, Alexandra Foubert‐Samier, Christine Brefel‐Courbon

**Affiliations:** ^1^ Department of Clinical Pharmacology and Neurosciences, Toulouse Parkinson Expert Centre, Toulouse NeuroToul Center of Excellence in Neurodegeneration (COEN), French NS‐Park/F‐CRIN Network University of Toulouse 3, CHU of Toulouse, INSERM Toulouse France; ^2^ Toulouse NeuroImaging Center University of Toulouse, Inserm, UPS Toulouse France; ^3^ MSA French Reference Center Univ. Hospital Toulouse Toulouse France; ^4^ CHU Bordeaux, Service de Neurologie des Maladies Neurodégénératives, CRMR AMS, IMNc Bordeaux France; ^5^ Univ. Bordeaux, CNRS, IMN UMR 5293 Bordeaux France; ^6^ AGO Formation Toulouse France; ^7^ UTEP, Univ. Hospital Toulouse Toulouse France

**Keywords:** multiple system atrophy, educational therapy, caregivers, quality of life

## Abstract

**Background:**

Multiple system atrophy (MSA) is a relentlessly progressive neurodegenerative disorder with no cure. While therapeutic education programs (TEPs) benefit many chronic conditions, data in MSA are limited.

**Objectives:**

To assess the feasibility and satisfaction of a mixed in‐person/virtual TEP for early‐stage MSA patients and caregivers.

**Methods:**

A six‐module, self‐paced TEP was co‐developed over 2 years by MSA healthcare professionals, patients, and education experts. A prospective pilot study enrolled recently diagnosed MSA patients (≤12 months) and caregivers. Primary outcomes were recruitment and retention rates. Secondary outcomes included disease progression (UMSARS I–II), health‐related quality of life (Hr‐QoL), compared to matched controls not enrolled in the TEP and patients’ satisfaction.

**Results:**

Fifteen patient‐caregiver pairs were enrolled. Recruitment and retention rates were 44% and 86%, respectively; 78% of modules were completed (least consulted was disease progression). Disease severity and Hr‐QoL worsened over 12 months, with no significant differences versus controls. Overall, 90% of participants were satisfied or very satisfied.

**Conclusions:**

Our pilot study shows that a mixed TEP for MSA patients and familial caregivers is feasible and able to engage them over time.

Multiple system atrophy (MSA) is an adult‐onset neurodegenerative disorder affecting the nigrostriatal system, cerebellum, pons, inferior olives and key brainstem and spinal cord nuclei involved in autonomic functions.^1^ Disease manifestations are complex, including parkinsonism, ataxia and autonomic failure. Symptoms progression is fast, and the survival is on average 6–9 years.^1^, ^2^ MSA leads to major disability, forcing patients and caregivers to massively adapt their environment and lifestyle.^3^ Treatment relies on symptomatic medications and non‐pharmacological approaches, preferentially delivered in a multidisciplinary care framework.^4^, ^5^ The announcement of an MSA diagnosis can be a shock for patients and their relatives, who often face a substantial workload and burden.^6^ Indeed, once the diagnosis is made, patients and caregivers must understand and cope with the symptoms, and navigate numerous questions and needs throughout the course of the disease.^7^ All these needs are not completely addressed by the current model of care, even in tertiary MSA centers. Therapeutic education programs (TEPs) have been proven to be effective in improving the management of several chronic conditions,^8^ including neurodegenerative diseases such as Parkinson's disease (PD).^9^, ^10^ TEP experience for MSA patients are scarce, with only one in‐presence program evaluated so far in a pilot study.^11^

To fill this gap, we developed an innovative mixed (virtual and in‐person) TEP for early MSA patients. Our project primarily aim was to test the feasibility of our TEP in a pilot study targeting recently diagnosed MSA patients and their caregivers. Program satisfaction has been also assessed as well as the effect of the program on health‐related quality of life (Hr‐QoL) over 1‐year follow‐up. For this latter analysis, we enriched the analysis relying on a comparison with matched MSA patients, not recruited into the TEP, belonging to our longitudinal cohort.

## Methods

### TEP Program Development

The development of the TEP took place between January 2020 and June 2022, and included the following: four virtual meetings, two brainstorming in‐person meetings and two meetings with ARAMISE, the French MSA patient association, to develop and analyze a patient survey and delineate program aims, 18 hybrid meetings to elaborate platform contents, and five meetings for platform graphical implementation. The steering committee in charge for the TEP development included: four neurologists, experts in MSA (WM, AFS, MF and APL), two MSA expert nurses (AR), one PD and TEP expert (CBC), three TEP experts (CT, EH and CM), one psychologist (CSDA) and one pedagogist (MCL). Platform contents were elaborated based on MSA healthcare professionals (HCP) expertise and MSA patients input. Indeed, a survey was conducted among 21 MSA patients and relative caregivers (16 couples recruited by the Toulouse and Bordeaux MSA Reference Center and five recruited by ARAMISE). MSA patients participating to the survey had a mean (SD) age and disease duration of 65 (6) years and 6 (4) years, respectively. The survey allowed to establish the foundations of the program, identify the needs for educational skills, and coping skills of MSA patients and familial caregivers at different stages of the disease. A qualitative analysis of the survey was carried out, indicating that patients and caregiver need to: (a) receive more information on the course and prognosis of the disease, its symptoms, causes, and available treatments; (b) be better trained on the support resources available (eg, they would like precise information on social services to better organize daily tasks related to disease management); (c) deal with psychological aspects, particularly their “depressive” symptoms and the suffering of caregivers; (d) have a caregiver support to maintain their own life projects.

Six modules (see dedicated paragraph), each one for specific patient and familial caregiver needs and skills were identified. According to the specific skills, the content of each module's part was prepared by a dedicated HCP expert (ie, the psychologist was in charge on the content on mood disorder, the neurologists for the content on MSA pathophysiology and pharmacological treatment, etc.). Once the content was finalized, its implementation on the platform was carried out through a collaborative effort involving MSA HCP, TEP experts, and two design and e‐learning platform specialists. The goal was to identify the most suitable digital tools and virtual formats to effectively deliver the objectives of each module. After the platform's graphic interface was drafted, two PD patients—both experienced in therapeutic education—reviewed all module contents and assessed usability, providing valuable feedback to enhance accessibility and user experience for patients.

### TEP Program Content

**Figure 1:**
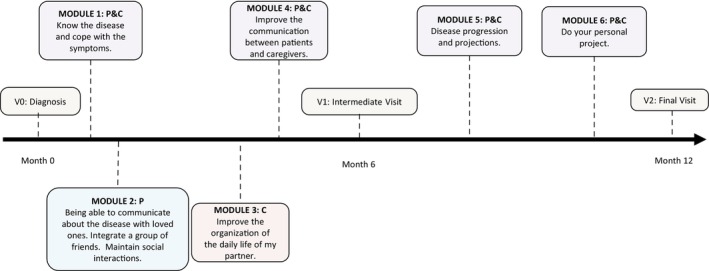
Multiple system atrophy‐therapeutic education program (MSA‐TEP) timeline. C, caregiver; P, patient.

The TEP was designed as a 12‐month program involving both patients and familial caregivers, with specific modules tailored separately for patients, caregivers, and both groups together. It followed a mixed model that included in‐person visits at baseline and at 12 months, along with continuous online activities such as learning modules, questionnaires, quizzes, videos, and interactive exercises. This mixed approach was adopted to ensure the program was feasible within patients’ schedules, comprehensive in its content, and accessible to those facing mobility challenges (Fig. 1).

The initial therapeutic assessment (V0) is essential for identifying patients’ needs before starting the education program. This assessment provides an opportunity to meet with patients and their familial caregivers, understand their lifestyles and specific needs. During this meeting patients and caregivers received instructions on platform function and use.

Participants needed to log in to the platform to complete the modules and they could decide to be enrolled in all the modules or pick‐up only some of them. The complete MSA‐TEP program includes (Fig. 1):

- Module 1 (for patients and caregivers in asynchronous remote): learning about the disease and its pathophysiology, recognizing symptoms, understanding both pharmacological and non‐pharmacological treatments, distinguishing between adverse events and disease symptoms, and knowing which healthcare professionals to contact when needed. It includes self‐paced online exercises for each topic. Patients and caregivers can access the platform at their convenience. The exercises are short and easy to complete, with the entire session taking approximately 45 min.
- Module 2 (for patients only): This is a virtual classroom involving three to four patients sharing their experiences. Through the use of photoexpression in order to work on patients’ perceptions and experiences, participants are encouraged to choose images and talk about their journey with the disease and share strategies for coping. The session has been pre‐designed to be facilitated by a psychologist or nurse. At the end, the option of psychological support can be discussed. The goal is to encourage open dialogue at home and help patients express their emotions to their loved ones.
- Module 3 (for caregivers only in asynchronous remote): This is an online exercise aimed at helping caregivers better organize the daily lives of their loved ones and prevent caregiver burnout. Caregivers can create a personalized schedule, explore social support options, and find resources to delegate care‐related tasks. At the end of the session, a downloadable document provides information on available social services.
- Module 4 (for patients and caregivers together): This virtual class brings together 3–4 patient‐caregiver pairs to explore whether the disease is a source of stress for caregivers and to identify strategies for managing it. Using photoexpression, participants are encouraged to share their feelings since the diagnosis. This session promotes mutual understanding and support. It has been pre‐structured for facilitation by a psychologist or nurse, and psychological support can be discussed afterward.
- Module 5: A short video featuring an expert MSA neurologist explains five key messages about the potential progression of the disease. After watching the video, a healthcare professional from the center will contact by phone participants to address any questions.
- Module 6 (for caregivers only in asynchronous remote): This online exercise supports caregivers in maintaining personal projects or hobbies. It allows them to compare their revised schedule (following Module 3) with their personal goals and plans.

### MSA Patients: Recruitment and Assessment

Inclusion criteria were a diagnosis of “clinically probable” or “clinically established” MSA^12^ parkinsonian (P) or cerebellar (C), made within the last 12 months, the presence of a familial caregiver (who lived with the patient or spent at least 3 h/day with him/her) and the cognitive ability to be enrolled within the program, according to physician's judgment. Exclusion criteria were red flags for alternative diagnosis. The lack of technical support (PC and webcam) was not considered an exclusion criterion as the MSA Center could offer a tablet, including a webcam to patients not having it (anyhow, it was not necessary as all participants had their own equipment).

### Outcome Measures

The primary outcome was the rate of eligible patients successfully enrolled in the study (‘recruitment rate’) and rate of patients completing 6 months (V1) and 12 months (V2) of the study protocol (“retention rate”) to test MSA‐TEP feasibility.

Secondary outcomes were:

- MSA‐QoL^13^, ^14^ score changes at V1 and V2 if compared to baseline (V0). The MSA‐QoL questionnaire is composed of three different subscales: motor (14 items), non‐motor (12 items) and emotional/social (14 items). MSA‐QoL total subscale scores were transformed so that they reflect a range of 0 to 100 (with higher scores indicating worse Hr‐QoL). Additionally, the questionnaire includes also a global visual analogue scale (VAS), where higher scores reflect better Hr‐QoL. Hr‐QoL changes were also compared to those of a MSA population (matched for time to diagnosis, sex and disease severity as assessed by UMSARS I + II^15^ and sex) extracted from the database of the French Reference Centre for MSA^3^, ^16^, ^17^ (current enrolment status = 830 MSA patients, with prospective once a year data on UMSARS and MSA‐QoL), who had not undergone TEP.
- Caregiver QoL changes were assessed by the Parkinsonism Carers QoL (PQoL Carer)^18^ at V1 and V2, compared to V0;
- Satisfaction of the patients as assessed by means of Likert scale questionnaires and a global satisfaction and benefit assessment on a 0–10 VAS (see Appendix S1), stratified for different aspects of the program, including content, ease of use and format.

Drop‐out reasons were also recorded.

### Statistical Analysis

For within‐subject comparisons of disease severity and Hr‐QoL scores across three time points—V0, V1 and V2 post TEP—the non‐parametric Friedman test was used. This test accounts for the repeated‐measures design without assuming normality. Where the Friedman test indicated a significant overall difference, post‐hoc pairwise comparisons were performed using the Nemenyi test. For comparisons between TEP patients and matched controls at two time points (baseline and follow‐up), the Wilcoxon signed‐rank test was used. Each TEP patient was matched to three MSA controls based on sex, age at inclusion (±5 years), disease duration (±12 months), baseline clinical severity (total UMSARS I‐II ±10) and Hr‐QoL (MSA total ± 10). Since each TEP patient had multiple controls, control scores were aggregated as the mean per matched set prior to paired testing. The follow‐up for controls was chosen as the closest visit occurring after the 12‐month mark post‐matching baseline. Data were analyzed using R (v4.0.4) and Python.

## Results

### Feasibility

Over June 2023 and February 2024 (9 months), we met 51 MSA patients at our in‐hospital or out‐hospital Department visits. Over 51, 34 had received the diagnosis within the last 12 months, of whom eight have been excluded due to the absence of a familial caregiver and 11 refused to be included (three were not interested in the program, three due to disease severity, and five due to other reasons, see Fig. 1).

**Figure 2:**
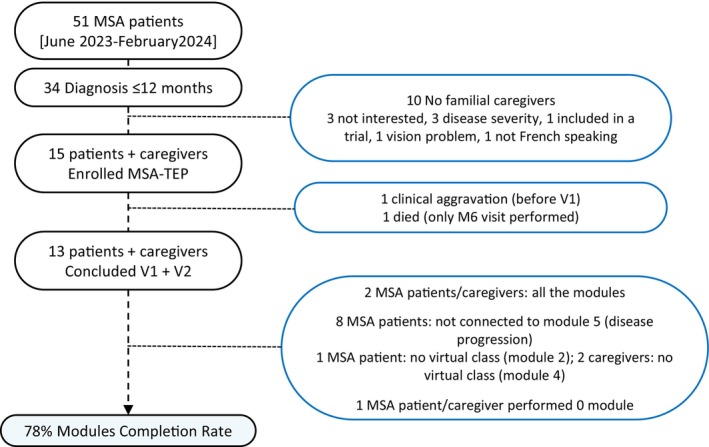
Flowchart for patients' inclusion and program competition.

We enrolled 15 MSA patients and relative caregivers, which resulted in a recruitment rate of 44% (15/34), including 1.6 patients/month. Clinical and demographic features of enrolled patients are detailed in Table 1. Overall, the mean (SD) age at onset and disease duration were 65 (6) years and 42.8 (17.1) months. Notably, no statistically significant differences were found between the 15 MSA patients included in the TEP program and the 19 not enrolled (see Flowchart) in terms of age, sex, or disease/diagnostic duration (data not shown in Tables), except for higher UMSARS I (27.8 ± 9.1 vs. 20.2 ± 6.5, *P* = 0.016) and UMSARS II (28.2 ± 9.4 vs. 19.3 ± 9.0, *P* = 0.012) scores in the TEP group. Thirteen patients and caregivers completed the program up to V2 visit, yielding a retention rate of 86%. Of these, one patient did not engage with any modules, resulting in a final sample of 12 patients for the analysis of TEP satisfaction, disease progression, and Hr‐QoL over time (Table 2). Detailed information on enrolment, dropout rates, and module completion are presented in Figure 2. Only two patients completed all modules, while one patient never accessed the platform due to mood‐related issues. The remaining ten patients partially participated in the program. Among these, Module 5 was the least accessed, with eight patients choosing not to receive further information about disease progression. Additionally, three patients did not attend the virtual classes (one missed both Modules 2 and 4, while two skipped the session involving the caregiver). Overall, 78% of the 78 total modules were completed. After TEP competition none asked for to have access to psychological support, but three patients were already followed by a psychologist before being enrolled into the program.

**TABLE 1:** Summary clinical and demographic data at baseline

Feature	Summary
Age at baseline (years)	65 ± 6 | 64 [61–69]
Mean ± SD | Median [Q1‐Q3]	13.8 ± 3 | 13 [13–16]
Years of education	
Mean ± SD | Median [Q1‐Q3]	
Diagnosis duration baseline (months) Mean ± SD | Median [Q1‐Q3]	7.2 ± 7.6 | 12 [0–12]
Disease duration considering motor symptom onset (months)	42.8 ± 17.1 | 36 [35–48]
Gender	10 Male, 5 Female
MSA‐P | MSA‐C	12 MSA‐P, 3 MSA‐C
Probable | Possible	10 MSA‐Prob, 5 MSA‐Poss
Caregiver Gender	7 Male, 8 Female
Caregiver Age (years)	64 ± 6 | 65 [63–67]
Mean ± SD | Median [Q1‐Q3]	

*Note*: Clinical and demographic features at baseline of enrolled MSA patients and relative caregivers.

**TABLE 2:** Summary disease severity scores at each evaluation

	V0 Mean ± SD Median [Q1‐Q3]	V1 Mean ± SD Median [Q1‐Q3]	V2 Mean ± SD Median [Q1‐Q3]	Friedman rank sum *P*‐value	V0‐V1	V1‐V2	V0‐V2
MSA QoL total score	64.5 ± 25.1 61 [46–78]	65.8 ± 17.5 71 [56–75]	75.9 ± 28.2 78 [64–89]	0.127	0.870	0.330	0.130
MSA QoL Motor	22.2 ± 8.1 22 [18–29]	25. ± 7.2 25 [22–30]	29.7 ± 10.7 30 [26–34]	**0.008**	0.755	0.064	**0.009**
MSA QoL Non‐motor	19.0 ± 9.2 16 [13–27]	19.7 ± 5.8 20 [16–24]	19.0 ± 8.3 17 [15–23]	0.455	0.500	0.990	0.560
MSA QoL Emotional	23.3 ± 12.8 21 [14–32]	20.9 ± 10.5 22 [16–25]	27.2 ± 12.5 29 [18–35]	0.121	0.870	0.130	0.330
MSA QoL VAS	48.3 ± 16.3 40 [40–53]	41.1 ± 9.4 40 [34–49]	39.1 ± 15.6 35 [28–50]	0.173	0.560	0.750	0.190
PDQ QoL Caregiver	39.0 ± 23.6 35 [22–51]	43.6 ± 30.1 43 [21–65]	49.4 ± 24.5 54 [31–72]	0.687	0.870	0.130	0.330
UMSARS I	19.3 ± 6.6 17 [16–20]	22.8 ± 6.5 21 [19–25]	25.9 ± 8.3 25 [21–32]	**0.008**	0.232	0.380	**0.009**
UMSARS II	18.6 ± 9.7 18 [12–21]	20.0 ± 9.7 18 [14–24]	26.5 ± 11.2 23 [19–36]	**0.028**	0.232	0.380	**0.009**
UMSARS I + II	37.5 ± 16.0 35 [26–41]	42 ± 15.5 40 [33–46]	52.6 ± 19.2 45 [41–68]	**0.020**	0.905	0.380	**0.009**

*Note*: Longitudinal scores progression for UMSARS, MSA Qol and PDQ Qol. N = 12. *p* values 〈 0.05 are indicated in bold values.

Abbreviation: MSA, multiple system atrophy.

### Disease Severity and Hr‐QoL Progression

Among the 12 patients who completed at least one module, significant worsening was observed in UMSARS I, UMSARS II, and the MSA‐QoL motor sub score from V0 to the V2 visit (*P* = 0.009). A non‐significant trend toward deterioration was observed at the 6‐month follow‐up for UMSARS I‐II, the MSA‐QoL total score, as well as the non‐motor/emotional MSA‐QoL sub scores and PQoL Carer at both V1 and V2 (Table 2). Conversely a non‐significant trend toward improvement was noted at the MSA‐QoL VAS. When comparing the progression of disease severity (UMSARS I + II) and Hr‐QoL scores over 1 year with those of a matched MSA population, no significant differences were found in the trajectory of either total scores or sub scores (Table 3).

**TABLE 3:** Summary clinical and demographic data: TEP cohort versus controls

Evaluation	Feature Mean ± SD | Median [Q1‐Q3] | %	TEP cohort	Controls rule‐based	Wilcoxon signed‐rank test
Baseline	Diagnosis duration (months)	7.2 ± 7.6 | 12 [0–12]	8.6 ± 8.4 | 73 [56–75]	‐
	Age at baseline (years)	65 ± 6 | 64 [61–69]	65 ± 6 | 64 [60–69]	‐
	Male (%)	67% male	67% male	‐
	MSA QoL Motor	22.2 ± 8.1 | 22 [18–29]	21.5 ± 9.3 | 21 [16–26]	‐
	MSA QoL Non‐motor	19.0 ± 9.2 | 16 [13–27]	16.0 ± 8.5 | 15 [10–22]	‐
	MSA QoL Emotional	23.3 ± 12.8 | 21 [14–32]	19.9 ± 11.1 | 18 [11–28]	‐
	MSA QoL Total score	64.5 ± 25.1 | 61 [46–78]	57.4 ± 22.5 | 50 [42–70]	‐
	UMSARS I	19.3 ± 6.6 | 17 [16–20]	19.5 ± 5.8 | 19 [17–22]	‐
	UMSARS II	18.6 ± 9.7 | 18 [12–21]	20.2 ± 6.9 | 20 [16–24]	‐
	UMSARS I + II	37.5 ± 16.0 | 35 [26–41]	39.6 ± 11.4 | 39 [32–44]	‐
12‐month Δ delta	Δ MSA QoL Motor	8.3 ± 7.2 | 9 [4–13]	7.9 ± 10.5 | 7 [2–16]	0.765
	Δ MSA QoL Non‐motor	2.8 ± 8.9 | 5 [−2–6]	1.2 ± 6.1 | 2 [−4–5]	0.450
	Δ MSA QoL Emotional	4.6 ± 10.3 | 6 [−5–13]	6.7 ± 13.1 | 9 [−3–18]	0.898
	Δ MSA QoL	15.6 ± 21.2 | 19 [−2–34]	15.3 ± 23.8 | 16 [−1–37]	0.966
	Δ UMSARS I	7.0 ± 7.6 | 7 [2–9]	5.5 ± 6.5 | 6 [3–7]	1.000
	Δ UMSARS II	7.7 ± 9.8 | 5 [0–12]	6.3 ± 6.9 | 6 [2–8]	1.000
	Δ UMSARS I + II	14.7 ± 16.2 | 7 [4–21]	13.9 ± 11.2 | 12 [7–15]	0.695

*Note*: Baseline and longitudinal comparison among MSA patients enrolled in the TEP versus a matched control group.

Abbreviation: MSA, multiple system atrophy.

### Satisfaction

**Figure 3:**
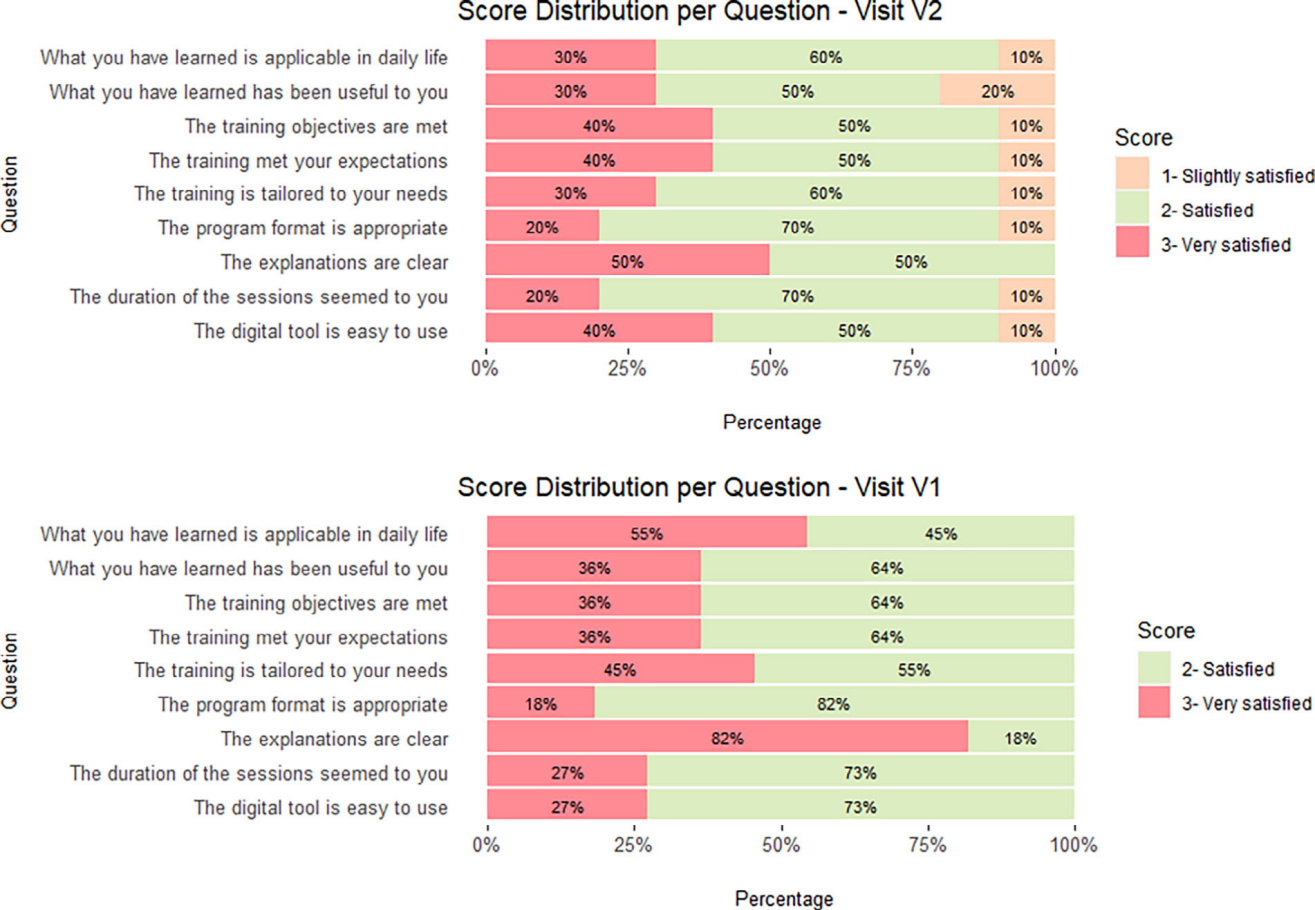
Distribution of response scores across nine items of satisfaction questionnaire for V1 (A) and V2 (B). Each horizontal stacked bar represents one item, with segments indicating the proportion of participants selecting each score. Percentages are displayed within each segment, and bars are normalized to 100% to facilitate comparison across items and visits.

Responses to individual items on the satisfaction questionnaire indicated a generally positive perception of the program, with approximately 90% of patients reporting being satisfied or very satisfied with its applicability, format, clarity of explanations, ease of use, and session duration (Fig. 3). Only 20% of patients rated what they had learned during the TEP as “slightly useful.” The mean (SD) ratings for overall program satisfaction and perceived benefit were 7.5 (0.8) and 7.1 (1.3), respectively, on a 0–10 VAS.

## Discussion

Our pilot study outlines the development process and demonstrates the feasibility and overall positive reception of a novel mixed‐format TEP, specifically designed for recently diagnosed MSA patients and familial caregivers. MSA is a rapidly progressive and complex neurodegenerative disorder that has a profound impact on the lives of patients and their families, often accompanied by significant unmet emotional and social needs. Our TEP focused on patients relatively close to their diagnosis, aiming to provide tailored educational interventions that address these challenges in a flexible and accessible way, including self‐paced learning and interactive virtual sessions. Additionally, our study aligns with the current trend advocating for integrated, patient‐centered care in neurodegenerative diseases.^19^ By incorporating patients’ and caregivers' voices into program development, we addressed unmet informational and emotional needs that are often overlooked in routine clinical encounters.

Regarding the observed cohort, demographic and clinical features are in line with those of several observational studies describing MSA.^16^, ^20^, ^21^ Thus, despite the inclusion criterion based on time since diagnosis, our study population appears representative of the broader MSA population, likely due to the typically long diagnostic delay from symptom onset. The recruitment rate (44%, including 1.6 patients/month) and high retention rate (86% at 1 year) suggest that such a program is both acceptable and logistically implementable within a tertiary care setting. Of note, we believe that the ability for patients and caregivers to engage with the modules at their own pace contributed to the program's adaptability. However, partial engagement with modules—particularly Module 5, which addresses disease progression—highlights a psychological barrier to discussing or confronting the anticipated trajectory of MSA. This module content merits to be further discussed with patients, to understand their expectation and feelings, as having a projection of their future was one of the main requests of the initial patients’ survey, but it clearly creates anxiety among the enrolled patients. The second least‐followed module was the one involving virtual classes, likely due to reluctance to share personal thoughts with other patients and caregivers. Nevertheless, it is important to note that learning about disease progression was identified as a key need in our initial patient survey. Furthermore, four patient‐caregiver pairs explicitly expressed their appreciation for the virtual classes in the free‐text responses of the satisfaction questionnaires. Taken together, these observations suggest that the program should include a degree of flexibility, allowing patients and caregivers to choose which modules to participate in. This would support a more personalized and needs‐based approach to the TEP.

High satisfaction scores across program content, format, and ease of use demonstrate that the TEP effectively met many participants’ expectations. However, 20% of participants rated what they learned as “slightly useful,” as they already had some information reading patients’ association website, suggesting room to enhance the personalization of the educational content. Heterogeneity in satisfactory scores can be related to differences in timing of delivery compared to disease duration and diagnosis, as well as variations in individual coping strategies and baseline knowledge about MSA. Indeed, if we look to disease and diagnosis duration they range between 3–4 years and 0–1 year, respectively, which can still reflect a likely possible heterogeneity in symptoms severity and understanding. As expected, despite the generally favorable reception of the MSA‐TEP, our longitudinal data show that disease severity (as measured by UMSARS I and II) and motor‐related QoL declined significantly over 12 months, consistent with the natural history of MSA. Comparisons with a matched MSA cohort not enrolled in the program revealed no significant differences in the trajectory of clinical or Hr‐QoL measures, suggesting that while the program may support coping and understanding, it does not alter the disease course neither patient Hr‐QoL. However, this aspect remains to be clarified, performed a study which is powered to demonstrate an effect on Hr‐QoL.

To the best of our knowledge, only one TEP for MSA patients has been previously proposed,^11^ though based on different design and content. Indeed, it consisted in eight in‐person group sessions of 60 min—each session on one disease aspect—over 6 months. As for our program, this Spanish in‐person TEP, reached optimal patients and caregivers’ satisfaction, despite no changes on motor scores neither Hr‐QoL that remained stable over 6 months.^11^ While in‐person educational sessions undoubtedly allow for more direct and effective communication, we chose a mixed‐format program to account for the motor disabilities common in MSA and to maximize flexibility for patients and caregivers. Indeed, although our MSA‐TEP was primarily based on virtual modules, it incorporated two in‐person visits, two virtual group sessions, and one follow‐up phone call. This design aimed to preserve human connection and ensure that participants did not feel isolated or left alone in front of a screen.

A future step of the program will be to refine the content based on patients and caregivers’ feedback. Initially, only two PD patients trained in therapeutic education reviewed the program, as no MSA patients with such expertise were available—no TEP existed for MSA in France and only one elsewhere. These PD patients helped assess the platform's clarity, accessibility, and usability. After the study, we gathered feedback from an MSA patient and her caregiver who had participated in the program. Their response was very positive, with two key suggestions: (1) target patients at very early stages of MSA, as earlier access would have been more beneficial, and (2) create a tiered caregiver entry, since not all content was relevant for those supporting patients with mild impairment. A few triage questions at the start could help tailor the experience. These suggestions will be discussed within the consortium, and offering the TEP early in the disease course appears especially valuable.

The main limitations of this study include the monocentric design, no caregiver control group, no data on education and socioeconomic background of not included patients (which would have helped better assess feasibility), the small sample size and potential self‐selection bias, as those more motivated or equipped to engage with a digital platform may have been overrepresented. Regarding the small sample size, it is important to consider that MSA is a rare and rapidly progressive condition. Our goal was to conduct a monocentric pilot feasibility study over a relatively short recruitment period (9 months), before offering it to a larger population of MSA patients and familial caregivers. Despite almost half of the eligible patients were included, we also recognize the relatively low recruitment rate, which prompts us to consider possible changes to the inclusion criteria to broaden the access to the program. As shown in Figure 1, the main reason for not inclusion was the absence of a familial caregiver. We are therefore considering the possibility of extending access to the platform to MSA patients who do not have a familial caregiver. As said, the study was not powered to detect subtle effects on Hr‐QoL or disease‐related outcomes, but to test the feasibility of an innovative MSA‐TEP. Additionally, although the program was designed to target recently diagnosed MSA patients, the typical long diagnostic delay resulted in the inclusion of individuals with relatively long symptom duration. Conversely, our TEP will likely be more beneficial once diagnostic timelines will be improved, allowing for earlier identification and truly early‐stage intervention—ideally at the onset of initial disease symptoms.

In conclusion, we developed an innovative mixed, virtual and in‐person, MSA‐TEP for both patients and caregivers. Our program results to be feasible with a good retention rate and patients/caregivers' engagement and satisfaction.

## Author Roles

(1) Research project: A. Conception, B. Organization, C. Execution; (2) Statistical Analysis: A. Design, B. Execution, C. Review and Critique; (3) Manuscript Preparation: A. Writing of the first draft, B. Review and Critique.

M.F.: 1A, 1B, 1C, 2C, 3A, 3B.

E.H.: 1A, 1B, 1C, 2C, 3B.

E.C.: 1C, 2C.

P.A.D.B.: 2B, 2C, 3A, 3B.

A.F.S, A.R., A.P.L.T., C.M., C.S.D.A., M.C.L., C.T.: 1A.

M.K.: 2B.

O.R.: 1A, 3B.

C.L., D.B.: 1C.

W.G.M.: 1A, 3B.

C.B.C.: 1A, 3B.

## Disclosures

**Ethical Compliance Statement:** All participants signed written informed consent after ethics approval was obtained by the Comité de Protection des Personnes (CPP) of the Sud Mediterranée III (RC31/22/0520 TEP@MS; national number: 2023‐A00340‐45). We confirm that we have read the Journal's position on issues involved in ethical publication and affirm that this work is consistent with those guidelines.

**Funding Sources and Conflicts of Interest:** The present work had been financed by a MSA Coalition GRANT 2022 and DGOS financement. The authors declare that there are no conflicts of interest relevant to this work.

**Financial Disclosures for the Previous 12 Months:** EH, EC, PADB, MCL, CT, MK, CL, AR, AP‐L‐T, CM, CSD'A, DB and AF‐S report no disclosures. MFreceived Honoraria to speak from AbbVie, ORKYN, and BIAL, consultancies from BIAL and LVL Medical; Grant from France Parkinson, HORIZON 2022 French Ministry of Health and MSA Coalition. CB‐C has received research grant from Association France Parkinson French Ministry of Health, and fees for lectures and consultancies from Aguettant, Orkyn, NHC, Zambon, Ellivie, everpharma and AbbVie. OR has acted as a scientific advisor for drug companies developing antiparkinsonian medications (Abbott, Abbvie, Acorda, Adamas, BIAL, Biogen, Boehringer‐Ingelheim, Cynapsus, GSK, Impax, Merck, Osmotica, Oxford‐Biomedica, Lundbeck, Novartis, Prexton, Servier, Sunovion, TEVA, UCB, Zambon). WGM received fees for consultancy from Lundbeck, Inhibikase, Koneksa and TreeFrog.

## Supporting information


**Appendix S1.** Satisfactory survey.

## Data Availability

The data that support the findings of this study are available on request from the corresponding author. The data are not publicly available due to privacy or ethical restrictions.
