# Supplementary material for: Development and Feasibility of a Mixed Virtual and in‐Presence Therapeutic Education Program for Early Multiple System Atrophy Patients
Source: Mov Disord Clin Pract. 2025 Aug 6;12(11):1900–8. doi: 10.1002/mdc3.70283 (PMC12625177; doi:10.1002/mdc3.70283)
Supplement: Supplementary file 1 — Appendix S1. Satisfactory survey. [file MDC3-12-1900-s001.docx]

**SATISFACTION QUESTIONNAIRE FOR THE TEP@MS THERAPEUTIC PROGRAM**

**Test completed:** 🞏1 Yes 🞏0 No

**Instructions:** To complete this questionnaire, simply circle the answer that best reflects your opinion.

## The training is tailored to your needs

Very satisfied 3 Satisfied 2 Slightly satisfied 1 Not at all satisfied 0

## The training objectives are met

Very satisfied 3 Satisfied 2 Slightly satisfied 1 Not at all satisfied 0

## The explanations are clear

Very satisfied 3 Satisfied 2 Slightly satisfied 1 Not at all satisfied 0

## What you have learned is applicable in daily life

Very satisfied 3 Satisfied 2 Slightly satisfied 1 Not at all satisfied 0

## What you have learned has been useful to you

Very satisfied 3 Satisfied 2 Slightly satisfied 1 Not at all satisfied 0

## The duration of the sessions seemed to you

Very satisfied 3 Satisfied 2 Slightly satisfied 1 Not at all satisfied 0

## The training met your expectations

Very satisfied 3 Satisfied 2 Slightly satisfied 1 Not at all satisfied 0

## The program format (in-person and online) is appropriate

Very satisfied 3 Satisfied 2 Slightly satisfied 1 Not at all satisfied 0

## The digital tool is easy to use

Very satisfied 3 Satisfied 2 Slightly satisfied 1 Not at all satisfied 0

**Please write in the boxes:**

| What did you appreciate the most? | What did you appreciate the least? |
| --- | --- |
|  |  |

**If 0 corresponds to no satisfaction and 10 to total satisfaction, how would you rate your satisfaction with the care you received as part of this educational program?**

# I_I_I

**Similarly, if 0 corresponds to no benefit and 10 to maximum benefit, how would you rate the benefit you think you gained from this support?**

# I_I_I

Comments and suggestions:

…………………………………………………………………………………………………………………………

…………………………………………………………………………………………………………………………

…………………………………………………………………………………………………………………………

…………………………………………………………………………………………………………………………

………………………………………………………………….................…
